# Employees' attitudes toward cancer, cancer survivors, and cancer survivors’ return to work

**DOI:** 10.1016/j.apjon.2023.100197

**Published:** 2023-02-04

**Authors:** Si Eun Lee, Eun Young Park

**Affiliations:** College of Nursing, Gachon University, Incheon, Republic of Korea

**Keywords:** Cancer survivor, Return to work, Social stigma, Public attitude, Neoplasms, Occupational groups

## Abstract

**Objective:**

This study aimed to evaluate employees' attitudes toward cancer, patients with cancer, and cancer survivors’ return to work.

**Methods:**

This study used a cross-sectional survey with online questionnaires to collect data during a 1-month period in April 2022. A stratified sampling method was used to select 237 participants. The data were analyzed using Pearson correlation coefficients and an independent t-test.

**Results:**

The following trends were observed regarding attitudes toward cancer and patients with cancer: impossibility of recovery: 9.00 ​± ​2.10 (4–16); stereotypes: 8.08 ​± ​2.12 (4–16); discrimination: 6.98 ​± ​2.26 (4–16); and financial instability: 7.37 ​± ​1.87 (3–12). Regarding public attitudes toward cancer survivors' return to work, the following results were confirmed: gender and living with family members/acquaintances who had survived cancer significantly impacted perceptions toward cancer survivors’ return to work.

For both variables (gender and job type), a significant difference was observed. Men had significantly higher negative perceptions of patients with cancer and their return to work than women, and there were significant differences between professional group and labor group. Moreover, participants living with cancer survivors (either among their family members or acquaintances) showed a significant difference in terms of attitudes toward cancer and patients with cancer and a greater recognition of such survivors’ return to the workplace.

**Conclusions:**

Despite a reduction in social stigma attached to cancer and cancer survivors, survivors may find returning to the workplace difficult. Public efforts and strategies are necessary for increasing awareness and reducing discrimination in society. This study's results could be used as basic data for establishing a social support system in the workplace and developing policies and educational programs to increase awareness about cancer survivors' issues.

## 1 Introduction

As cancer incidence has increased and 5-year relative survival rates have improved, the number of cancer survivors returning to daily life has been increasing rapidly. Helping survivors return to work after their cancer diagnosis has become an important issue, not only with regard to the survivors themselves but also with certain associated socioeconomic issues.^1^^,^^2^ For cancer survivors, continuing to work or returning to work is an important factor in constructing their self-identity, quality of life, social connections, and activities of daily living^3^^,^^4^; it helps to restore individual self-esteem by providing a fixed income source, which, in turn, allows the patient with cancer to achieve economic stability.^5^ Thus, it plays an important part in the processes of treatment and recovery.^6^

Cancer survivors' capability to return to work and lead a successful life depends on the understanding and active acceptance shown to them by their organizational members^7^; furthermore, factors such as mutual respect among coworkers and whether they are emotionally supportive have been shown to have an effect on successful return to work.^8^ However, doubts about cancer survivors’ capability to perform their roles properly, lack of emotional support from peers, and lack of a support system in their organization make it more difficult for cancer survivors to adapt to the organization.^9^ Some cancer survivors may even experience conflict and hurt from their interactions with their coworkers.^10^ The potential to lose their jobs or be forced to change jobs because of the social stigma they face as cancer survivors is strong.^11^

Coworkers’ attitudes toward cancer, cancer survivors, and their return to work can have a significant influence on their successful return to work; therefore, the degree of acceptance is important. Reportedly, such perceptions have been an important factor in establishing workplace social support systems for cancer survivors,^9^ and they can contribute toward improving the organizational health of both cancer survivors and their coworkers^12^, ^13^, ^14^.

Several studies have reported that factors such as socioeconomic status, level of education, and manual labor make it difficult for cancer survivors to return to work; much information has already been provided through occupational, medical, physical, psychological, and educational interventions in this regard.^15^^,^^16^ However, in South Korea (henceforth Korea), cancer survivors are able to access a limited return-to-work social support system,^17^ and many cancer survivors are, therefore, reluctant to continue any work they have undertaken or return to work.^18^^,^^19^ Supporting business establishments may also fail to provide support and consideration.^9^^,^^20^ A previous study that examined public perceptions regarding cancer survivors' return to work in Korea^21^ focused only on middle-aged, socially active individuals and, therefore, did not assess such attitudes among people in their 20s and 30s, who account for 38% of the productive population (ages: 15–64 years) in Korea.^22^ The previous study^10^ that investigated office workers’ perceptions regarding cancer and cancer survivors, there are limitations in that the data are from 10 years ago, the proportion of retirees is high, and the distribution among occupations is concentrated on blue-collar workers, making it difficult to fully represent the perceptions of the current workforce.

As noted above, studies to date have not found an association between cancer survivors' return to work and general attitudes toward cancer and cancer survivors.

Therefore, this study aims to investigate the general attitudes of workers toward cancer and cancer survivors and their perceptions of cancer survivors returning to work, specifically according to occupation type. Moreover, this study aims to explain the relationship between related variables.

## 2 Methods

### 2.1 Study design

This cross-sectional survey-based study investigated attitudes toward cancer and cancer survivors as well as perceptions about cancer survivors' return to work.

### 2.2 Participants and data collection

The participants were aged 20 years or older who were working in Korea at the time of the survey. Data were collected through an online survey, as COVID-19 made face-to-face surveys difficult. Data collection was conducted by e-mail, mobile, and SNS message through a survey agency (Korea Research, Korean Research Co.) for a month from April 1, 2022, to April 30, 2022. The questionnaire was sent to 3179 people who met the inclusion criteria of this study among the panel members who joined the company.

The appropriate number of participants in this study was 200 according to G-power 3.1.9.2, significance level 0.05, effect size 0.25, power 80%, ANOVA: fixed effects, omnibus, one-way. However, to compare the differences between the four occupational groups and ensure an even age distribution by occupational group, data collection was closed when the distribution of personnel by all occupational groups and age groups was satisfied. A total of 364 respondents participated, excluding unfaithful responses, and after distributing the number of participants in each group in proportion, the data of the final 237 respondents were used for analysis.

### 2.3 Instruments

#### 2.3.1 Attitudes toward cancer and patients with cancer

This current study assessed perceptions regarding cancer and cancer survivors using a questionnaire modified by Park et al.^29^ (with the author's approval) based on the “Attitude of cancer and cancer patients” questionnaire of Cho et al.^10^ Cho et al’s^10^ original questionnaire included four items on impossibility of recovery, four items on stereotypes of patients with cancer, four items on discrimination against patients with cancer, and three items on nondisclosure of cancer diagnosis. Park et al.^29^ modified the tool and added three items on financial vulnerability. Each item is assessed using a 4-point Likert scale, with a higher score indicating a more negative attitude and total scores ranging from 18 to 72. The reliability in the study of Cho et al.^10^ was indicated by a Cronbach α of 0.79; in this study, Cronbach α was 0.895. As for the reliability of subdomains, impossibility of recovery was 0.758, stereotypes of patients with cancer was 0.763, discrimination against patient with cancer was 0.78, financial Instability was 0.778, and not open to the public was 0.889.

#### 2.3.2 Public attitudes toward cancer survivors’ return to work

To evaluate perceptions regarding cancer survivors' return to work, this study used a questionnaire developed by Shim et al.^21^ (with the author's approval). It included two areas: perceptions regarding reinstatement (13 items) and acceptance toward return to work (6 items); each item was assessed using a 4-point Likert scale. Scores for perceptions regarding reinstatement ranged from 13 to 52, and higher scores indicated negative perceptions. Acceptance of cancer survivors' return to work included six items, with the score ranging from 6 to 24; a lower score indicated a more negative attitude toward survivors' return to work. In the study by Shim et al, ^21^ the Cronbach α was 0.84; in the current study, the Cronbach α was 0.851. The subdomain of perceptions toward cancer survivors returning to work was 0.915 and of acceptance of cancer survivors returning to work was 0.82.

### 2.4 Data analysis

The general characteristics of the study participants, their perceptions regarding cancer and cancer survivors and their perceptions regarding cancer survivors' return to work were analyzed using mean, percentage, and standard deviation. The correlations between their perceptions regarding cancer and cancer survivors and perceptions regarding cancer survivors' return to work were analyzed using Pearson correlation coefficients. The differences were analyzed using a t-test and ANOVA, followed by Scheffe test as a post hoc method. All statistical tests were two-sided, and a *P*-value <0.05 was considered statistically significant.

### 2.5 Ethical considerations

The study was approved by the Institutional Review Board of the Gachon University (IRB No. 1044396-202201-HR-017). All participants provided written informed consent.

## 3 Results

### 3.1 General characteristics

The participants included 120 men and 117 women. Considering occupational distribution, 24.5% were professional/technical workers, 26.6% were office/administrative workers, 24.5% were sales/service workers, and 24.5% were production/transport/labor workers. The average age was 41.83 years, and it was distributed from 20 to 50 years or older. As for work type, full-time workers formed the largest section of the participants, and by position, “employee level” workers formed the largest section with 48.5%. Regarding education, 67.9% of the participants had a college degree or higher qualification, and the highest monthly income bracket ranged from 1500–3000 USD. The percentage of participants who had a history of cancer was 4.36% (Table 1).

**Table 1:** Characteristics of study participants (*n* ​= ​237).

Characteristics		*n* (%)
Gender	Male	120 (50.6)
	Female	117 (49.4)
Age, years	20–29	58 (24.5)
	30–39	59 (24.9)
	40–49	58 (24.5)
	> 50	62 (26.2)
	Mean ± SD	41.83 ​± ​11.44
Occupation	Professional/technical	58 (24.5)
	Office/administrative	63 (26.6)
	Sales/Service worker	58 (24.5)
	Production/Transportation/Labor	58 (24.5)
Years of working	< 1	17 (7.2)
	1–3	48 (20.3)
	3–5	21 (8.9)
	5–10	59 (24.9)
	> 10	92 (38.8)
Shift pattern	Regular	163 (68.8)
	Contract	40 (16.9)
	Daily/Part-time	16 (6.8)
	Self-employment/employer	18 (7.6)
Position	Staff	115 (48.5)
	Assistant manager	40 (16.9)
	Manager	23 (9.7)
	Deputy general manager/General manager	32 (13.5)
	Director	9 (3.8)
	Others (self-employment/employer)	18 (7.6)
Staff numbers	1–49	116 (48.9)
	50–299	65 (27.4)
	300–4999	56 (23.6)
Workplace	Indoor job	190 (80.2)
	Outside job	47 (19.8)
Monthly household income (USD)	< 1500	45 (19.0)
	1500–3000	142 (59.9)
	> 3000	50 (21.1)
Education	Less than middle school	2 (0.8)
	High school	74 (31.2)
	College/university	137 (57.8)
	Higher than graduate school	24 (10.1)
Cancer history	Yes	11 (4.6)
	No	226 (95.4)
Living with cancer survivors among family or acquaintances	Yes	116 (48.9)
	No	121 (51.1)
Paths for obtaining cancer-related information	TV/Radio	99 (41.8)
	Internet	103 (43.5)
	YouTube	30 (12.7)
	Book	2 (0.8)
	Others	3 (1.3)

### 3.2 Attitudes regarding cancer and patients with cancer: public attitudes regarding cancer survivors’ return to work

Among the questions regarding participants' perceptions of cancer and cancer survivors, the item that elicited the most negative perception was “Patients with cancer cannot guarantee the safety of their workplace because of their cancer” (2.53 ​± ​0.78). Further, the item that elicited the least negative perception was “I would avoid working with people who have cancer” (1.54 ​± ​0.65) (Table 2).

**Table 2:** Attitudes toward cancer and patients with cancer (*n* ​= ​237).

Subdomain	Item	*n* (%)				Mean ​± ​SD
		**Strongly disagree (1)**	**Disagree (2)**	**Agree (3)**	**Strongly agree (4)**	
Impossibility of recovery	It is impossible to treat cancer regardless of highly developed medical science	12 (5.1)	104 (43.9)	106 (44.7)	15 (6.3)	2.52 ​± ​0.69
	It is very difficult to be healthy again once a person is diagnosed with cancer	11 (4.6)	136 (57.4)	82 (34.6)	8 (3.4)	2.37 ​± ​0.63
	Patients with cancer would not be socially active once diagnosed with cancer	63 (26.6)	130 (54.9)	40 (16.9)	4 (1.7)	1.94 ​± ​0.71
	The ability of patients with cancer to perform tasks at the workplace may decrease even after successful cancer treatment	41 (17.3)	118 (49.8)	73 (30.8)	5 (2.1)	2.18 ​± ​0.73
						9.00 (16) ​± ​2.10
Stereotypes of patients with cancer	Patients with cancer are easily recognized through their looks	69 (29.1)	139 (58.6)	26 (11.0)	3 (1.3)	1.84 ​± ​0.65
	Patients with cancer would have a difficult time having sexual intimacy	38 (16.0)	149 (62.9)	44 (18.6)	6 (2.5)	2.08 ​± ​0.67
	Patients with cancer deserve to be protected in society.	36 (15.2)	102 (43.0)	87 (36.7)	12 (5.1)	2.32 ​± ​0.79
	Patients with cancer would not be able to make contributions to society	68 (28.7)	142 (59.9)	23 (9.7)	4 (1.7)	1.84 ​± ​0.65
						8.08 (16) ​± ​2.12
Discrimination against patient with cancer	I feel uncomfortable when I am with patients with cancer	98 (41.4)	102 (43.0)	32 (13.5)	5 (2.1)	1.76 ​± ​0.76
	I tend to avoid interacting with neighbors who have cancer	128 (54.0)	89 (37.6)	19 (8.0)	1 (0.4)	1.55 ​± ​0.66
	I would avoid marrying people whose family members have cancer	56 (23.6)	104 (43.9)	66 (27.8)	11 (4.6)	2.14 ​± ​0.83
	I would avoid working with people who have cancer	125 (52.7)	101 (42.6)	6 (2.5)	5 (2.1)	1.54 ​± ​0.65
						6.98 (16) ​± ​2.26
Financial Instability	Patients with cancer will suffer from low self-esteem due to economic difficulties	17 (7.2)	94 (39.7)	114 (48.1)	12 (5.1)	2.51 ​± ​0.70
	Patients with cancer can't guarantee the safety of their workplace because of cancer	24 (10.1)	82 (34.6)	113 (47.7)	18 (7.6)	2.53 ​± ​0.78
	Patients with cancer may be affected by personal relationships due to economic difficulties	34 (14.3)	96 (40.5)	99 (41.8)	8 (3.4)	2.34 ​± ​0.76
						7.37 (12) ​± ​1.87
Not open to the public	If I get cancer, I don't want to tell my family about cancer	53 (22.4)	112 (47.3)	57 (24.1)	15 (6.3)	2.14 ​± ​0.84
	If I get cancer, I don't want to tell my neighbors or friends about cancer	37 (15.6)	99 (41.8)	81 (34.2)	20 (8.4)	2.35 ​± ​0.84
	If I get cancer, I don't want to tell my coworker about cancer	29 (12.2)	106 (44.7)	83 (35.0)	19 (8.0)	2.39 ​± ​0.80
						6.88 (12) ​± ​2.24

						38.33 (72) ​± ​7.91

The average score for reinstatement recognition was 27.81 points (SD ​= ​6.59). The average score for reinstatement acceptance was 12.71 points (SD ​= ​3.15). With regard to reinstatement perceptions, the item with the highest negative recognition level was “Lower ability of patients with cancer to work due to pain after treatment” with a score of 2.65 ​± ​0.66 (Table 3).

**Table 3:** Public attitudes toward cancer survivors regarding return to work (*n* ​= ​237).

Subdomain	Item	*n* (%)				Mean ​± ​SD
		Strongly disagree (1)	Disagree (2)	Agree (3)	Strongly agree (4)	
Perceptions towards cancer survivors returning to work^a^	Lower ability of patients with cancer to work due to pain after treatment	14 (5.9)	65 (27.4)	147 (62.0)	11 (4.6)	2.65 ​± ​0.66
	Poor performance of patients with cancer due to lack of concentration and memory loss	11 (4.6)	110 (46.4)	109 (46.0)	7 (3.0)	2.47 ​± ​0.63
	Trouble working due to post-treatment depression in patients with cancer	22 (9.3)	90 (38.0)	110 (46.4)	15 (6.3)	2.50 ​± ​0.75
	Feelings of discomfort when seeing wigs and hats on patients with cancer during work	75 (31.6)	124 (52.3)	35 (14.8)	3 (1.3)	1.86 ​± ​0.70
	Since patients with cancer feel weak, their coworkers may feel weak as well	52 (21.9)	133 (56.1)	46 (19.4)	6 (2.5)	2.03 ​± ​0.72
	Less confidence at work among patients with cancer	45 (19.0)	109 (46.0)	76 (32.1)	7 (3.0)	2.19 ​± ​0.77
	Even though most patients with cancer want to work, there might not be much work available for them	38 (16.0)	124 (52.3)	62 (26.2)	13 (5.5)	2.21 ​± ​0.77
	Since patients with cancer are less competitive, they should depend on their families, instead of re-entering society	61 (25.7)	139 (58.6)	29 (12.2)	8 (3.4)	1.93 ​± ​0.72
	Patients with cancer should retire early	68 (28.7)	131 (55.3)	29 (12.2)	9 (3.8)	1.91 ​± ​0.75
	Companies should hire healthy new employees rather than experienced patients with cancer	40 (16.9)	126 (53.2)	62 (26.2)	9 (3.8)	2.17 ​± ​0.75
	Companies should consider patients with cancer first during layoffs	77 (32.5)	127 (53.6)	31 (13.1)	2 (0.8)	1.82 ​± ​0.68
	I feel insecure about the job abilities of coworkers who have cancer	49 (20.7)	142 (59.9)	42 (17.7)	4 (1.7)	2.00 ​± ​0.67
	Employees with cancer should be considerate of their coworkers by not attending corporate events	54 (22.8)	121 (51.1)	53 (22.4)	9 (3.8)	2.07 ​± ​0.78
						27.81 (52)±6.59
Acceptance of cancer survivors returning to work^b^	A work environment should be created such that coworkers with cancer can work as ordinary employees	15 (6.3)	35 (14.8)	139 (58.6)	48 (20.3)	2.93 ​± ​0.78
	I agree on a reduction in work hours for coworkers with cancer at) the same pay	14 (5.9)	84 (35.4)	118 (49.8)	21 (8.9)	2.62 ​± ​0.73
	I agree that coworkers with cancer should be entitled to special long-term vacations over 6-month periods	12 (5.1)	43 (18.1)	133 (56.1)	49 (20.7)	2.92 ​± ​0.77
	I agree that coworkers with cancer should be entitled to promotions	9 (3.8)	37 (15.6)	152 (64.1)	39 (16.5)	2.93 ​± ​0.69
	Patients with cancer should be given equal promotional opportunities	4 (1.7)	21 (8.9)	155 (65.4)	57 (24.1)	3.12 ​± ​0.62
	A resting place should be provided for coworkers with cancer in the workplace	15 (6.3)	57 (24.1)	134 (56.5)	31 (13.1)	2.76 ​± ​0.76
						12.71 (24)±3.15

a Higher scores are negative.

b Lower scores are negative.

### 3.3 Correlations between attitudes toward cancer and patients with cancer and perceptions regarding cancer survivors’ return to work

Among the subdomains of perceptions regarding cancer and cancer survivors, recovery, prejudice, and discrimination did not show a significant relationship with acceptance of cancer survivors' return to work. Within the subdomains of “financial instability” and “not open to the public,” there was a significant relationship between recognition of cancer survivors' return to work and acceptance toward their return to work. There was no significant relationship between perceptions regarding reinstatement and acceptance of cancer survivors’ return to work (Table 4).

**Table 4:** Correlation between cancer and perceptions of cancer survivors, and perceptions of cancer survivors returning to work (*n* ​= ​237).

	Attitudes toward cancer and patients with cancer						Public attitudes toward cancer survivors regarding return to work		
	Recovery	Stereotype	Discrimination	Financial	Not open	Total	Perception	Acceptance	Total
Attitudes toward cancer and patients with cancer									
Recovery	1								
Stereotype	0.566∗∗∗ (< 0.001)	1							
Discrimination	0.500∗∗∗ (< 0.001)	0.653∗∗∗ (< 0.001)	1						
Financial	0.399∗∗∗ (< 0.001)	0.464∗∗∗ (< 0.001)	0.411∗∗∗ (< 0.001)	1					
Not open	0.301∗∗∗ (< 0.001)	0.386∗∗∗ (< 0.001)	0.346∗∗∗ (< 0.001)	0.430∗∗∗ (< 0.001)	1				
Total	0.740∗∗∗ (< 0.001)	0.824∗∗∗ (< 0.001)	0.789∗∗∗ (< 0.001)	0.706∗∗∗ (< 0.001)	0.668∗∗∗ (< 0.001)	1			
Public attitudes toward cancer survivors regarding return to work									
Perception	0.494∗∗∗ (< 0.001)	0.581∗∗∗ (< 0.001)	0.668∗∗∗ (< 0.001)	0.500∗∗∗ (< 0.001)	0.356∗∗∗ (< 0.001)	0.697∗∗∗ (< 0.001)	1		
Acceptance	−0.052 (0.426)	0.043 (0.511)	−0.077 (0.236)	0.196∗ (< 0.05)	0.189∗ (< 0.5)	0.76 (0.246)	−0.57 (0.384)	1	

∗*P* < 0.05, ∗∗*P* < 0.01, ∗∗∗*P* < 0.001.

### 3.4 Attitudes toward cancer and cancer survivors based on general characteristics

Among the subdomains, there was a significant difference in financial instability by occupational group (*P* ​< ​0.05). Depending on whether participant's lived with cancer survivors (family or acquaintances), the subdomains of stereotypes (*t* ​= ​−2.688, *P* ​< ​0.001), discrimination (*t* ​= ​−2.528, *P* ​< ​0.05), and nondisclosure (*t* ​= ​−2.084, *P* ​< ​0.05) significantly differed. Among the four occupation groups, professional and technical workers scored the lowest, with an average of 7.03 points (SD ​= ​1.95), and production, transportation, and labor jobs scored an average of 7.88 points (SD ​= ​1.76), thus demonstrating the highest negative perception (Table 5).

**Table 5:** Attitudes toward cancer and patients with cancer according to general characteristics (*n* ​= ​237).

		*n* (%)	Recovery (Mean ± SD)	*t/F* (*P*)	Stereotype (Mean ± SD)	*t/F* (*P*)	Discrimination (Mean ± SD)	*t/F* (*P*)	Financial (Mean ± SD)	*t/F (*P*)*	Not open (Mean ± SD)	*t/F (*P*)*	Total (Mean ± SD)	*t/F (*P*)*
Gender	Male	120 (50.6)	9.16 ​± ​2.05	1.143 (0.254)	8.52 ​± ​2.29	3.274∗∗ (< 0.01)	7.24 ​± ​2.48	1.760 (0.080)	7.63 ​± ​1.70	2.129∗ (<0.05)	7.15 ​± ​2.18	1.840 (0.067)	39.70 ​± ​8.31	2.720∗∗ (< 0.01)
	Female	117 (49.4)	8.85 ​± ​2.15		7.63 ​± ​1.83		6.73 ​± ​1.99		7.12 ​± ​2.01		6.62 ​± ​2.29		36.94 ​± ​7.26	
Age (yrs)	20–29	58 (24.5)	9.28 ​± ​2.04	1.381 (0.249)	7.79 ​± ​2.03	1.236 (0.297)	6.76 ​± ​2.36	0.347 (0.791)	7.10 ​± ​1.71	0.720 (0.541)	6.62 ​± ​2.25	1.550 (0.202)	37.55 ​± ​7.88	0.910 (0.437)
	30–39	59 (24.9)	9.29 ​± ​2.13		8.51 ​± ​2.47		7.07 ​± ​2.57		7.44 ​± ​2.11		7.15 ​± ​2.41		39.46 ​± ​8.54	
	40–49	58 (24.5)	8.67 ​± ​2.33		7.95 ​± ​2.09		6.95 ​± ​2.22		7.36 ​± ​1.88		6.52 ​± ​1.91		37.45 ​± ​7.43	
	> 50	62 (26.2)	8.79 ​± ​1.88		8.06 ​± ​1.84		7.16 ​± ​1.92		7.60 ​± ​1.77		7.23 ​± ​2.34		38.84 ​± ​7.78	
Occupation	Professional/technical	58 (24.5)	8.74 ​± ​2.21	2.349 (0.073)	8.00 ​± ​2.04	1.401 (0.243)	6.76 ​± ​2.28	0.847 (0.469)	7.03 ​± ​1.95	2.805∗ (< 0.05)	6.79 ​± ​2.29	0.561 (0.641)	37.33 ​± ​8.01	1.709 (0.166)
	Office/administrative	63 (26.6)	8.59 ​± ​1.86		7.79 ​± ​1.85		7.02 ​± ​2.16		7.08 ​± ​1.73		6.95 ​± ​2.11		37.43 ​± ​7.03	
	Sales/Service worker	58 (24.5)	9.45 ​± ​2.05		8.00 ​± ​2.35		6.81 ​± ​2.47		7.55 ​± ​1.97		6.64 ​± ​2.30		38.45 ​± ​8.55	
	Production/Transportation/Labor	58 (24.5)	9.28 ​± ​2.21		8.55 ​± ​2.20		7.36 ​± ​2.14		7.88 ​± ​1.76		7.16 ​± ​2.32		40.22 ​± ​7.93	
Years of working	< 1	17 (7.2)	9.82 ​± ​1.70	1.965 (0.101)	7.82 ​± ​1.38	1.447 (0.219)	6.53 ​± ​1.87	0.421 (0.793)	8.18 ​± ​1.38	1.076 (0.369)	7.18 ​± ​2.35	0.452 (0.771)	39.53 ​± ​5.21	0.380 (0.823)
	1–3	48 (20.3)	9.17 ​± ​2.40		7.65 ​± ​2.24		6.75 ​± ​2.40		7.25 ​± ​2.07		6.71 ​± ​2.48		37.52 ​± ​8.52	
	3–5	21 (8.9)	9.62 ​± ​2.01		8.67 ​± ​2.03		7.19 ​± ​2.60		7.10 ​± ​1.81		6.48 ​± ​1.81		39.05 ​± ​7.83	
	5–10	59 (24.9)	9.02 ​± ​1.91		8.44 ​± ​2.51		7.12 ​± ​2.55		7.24 ​± ​1.94		7.10 ​± ​2.38		38.92 ​± ​8.82	
	> 10	92 (38.8)	8.62 ​± ​2.10		7.99 ​± ​1.88		7.07 ​± ​1.99		7.46 ​± ​1.80		6.88 ​± ​2.12		38.01 ​± ​7.49	
Shift pattern	Regular	163 (68.8)	8.82 ​± ​2.07	2.784 (0.042)∗	8.00 ​± ​2.17	0.562 (0.641)	7.08 ​± ​2.34	0.289 (0.834)	7.20 ​± ​1.86	1.810 (0.146)	6.80 ​± ​2.23	0.416 (0.742)	37.89 ​± ​8.04	0.800 (0.495)
	Contract	40 (16.9)	9.28 ​± ​1.66		8.48 ​± ​2.06		6.78 ​± ​2.11		7.85 ​± ​1.86		7.20 ​± ​2.49		39.58 ​± ​7.65	
	Daily/Part-time	16 (6.8)	10.31 ​± ​2.41		8.06 ​± ​1.84		6.81 ​± ​2.01		7.88 ​± ​2.13		7.13 ​± ​2.36		40.19 ​± ​7.76	
	Self-employment/employer	18 (7.6)	8.94 ​± ​2.62		7.94 ​± ​2.04		6.78 ​± ​2.21		7.56 ​± ​1.65		6.78 ​± ​1.83		38.00 ​± ​7.55	
Position	Staff	115 (48.5)	9.26 ​± ​2.07	0.939 (0.457)	7.99 ​± ​2.14	0.666 (0.650)	6.94 ​± ​2.33	0.535 (0.750)	7.54 ​± ​2.03	1.109 (0.356)	6.90 ​± ​2.44	0.528 (0.755)	38.63 ​± ​8.23	0.257 (0.936)
	Assistant manager	40 (16.9)	8.95 ​± ​1.99		8.58 ​± ​2.41		7.48 ​± ​2.59		6.88 ​± ​1.65		7.15 ​± ​2.05		39.03 ​± ​8.17	
	Manager	23 (9.7)	8.39 ​± ​1.53		7.96 ​± ​1.87		6.74 ​± ​1.94		7.04 ​± ​2.03		7.00 ​± ​2.04		37.13 ​± ​7.00	
	Deputy General manager/General manager	32 (13.5)	8.78 ​± ​2.30		8.13 ​± ​1.86		6.78 ​± ​1.88		7.66 ​± ​1.45		6.38 ​± ​2.11		37.72 ​± ​7.30	
	Director	9 (3.8)	8.44 ​± ​2.51		7.44 ​± ​2.30		7.22 ​± ​2.17		7.11 ​± ​1.90		7.33 ​± ​2.55		37.56 ​± ​9.28	
	Others (self-employment/employer)	18 (7.6)	8.94 ​± ​2.62		7.94 ​± ​2.04		6.78 ​± ​2.21		7.56 ​± ​1.65		6.78 ​± ​1.83		38.00 ​± ​7.55	
Staff numbers	1–49	116 (48.9)	9.03 ​± ​2.20	0.246 (0.782)	7.93 ​± ​2.39	1.141 (0.321)	6.93 ​± ​2.29	0.071 (0.931)	7.34 ​± ​1.86	0.677 (0.509)	6.89 ​± ​2.26	0.893 (0.411)	38.11 ​± ​8.40	0.727 (0.484)
	50–299	65 (27.4)	8.86 ​± ​2.16		8.03 ​± ​1.98		7.03 ​± ​2.38		7.25 ​± ​1.97		6.63 ​± ​2.57		37.80 ​± ​8.34	
	300–4999	56 (23.6)	9.13 ​± ​1.84		8.45 ​± ​1.62		7.05 ​± ​2.09		7.63 ​± ​1.77		7.18 ​± ​1.78		39.43 ​± ​6.21	
Workplace	Indoor job	190 (80.2)	8.88 ​± ​2.14	−1.852 (0.065)	7.97 ​± ​2.22	−1.559 (0.120)	6.95 ​± ​2.32	−0.474 (0.636)	7.26 ​± ​1.85	−1.940 (0.054)	6.88 ​± ​2.30	−0.026 (0.980)	37.95 ​± ​8.14	−1.509 (0.133)
	Outside job	47 (19.8)	9.51 ​± ​1.88		8.51 ​± ​1.60		7.13 ​± ​2.03		7.85 ​± ​1.90		6.89 ​± ​2.03		39.89 ​± ​6.80	
Monthly household income (USD)	< 1500	45 (19.0)	9.64 ​± ​2.20	2.640 (0.073)	8.16 ​± ​2.12	0.270 (0.763)	6.96 ​± ​2.26	0.007 (0.993)	7.62 ​± ​1.86	1.443 (0.238)	7.11 ​± ​2.43	0.278 (0.758)	39.49 ​± ​8.25	0.739 (0.479)
	1500–3000	142 (59.9)	8.83 ​± ​1.98		8.00 ​± ​2.18		7.00 ​± ​2.29		7.21 ​± ​1.84		6.84 ​± ​2.24		37.88 ​± ​7.85	
	> 3000	50 (21.1)	8.92 ​± ​2.29		8.24 ​± ​1.99		6.98 ​± ​2.23		7.64 ​± ​1.94		6.82 ​± ​2.14		38.60 ​± ​7.83	
Education	Less than middle school	2 (0.8)	9.50 ​± ​0.71	1.216 (0.305)	8.50 ​± ​0.71	0.093 (0.964)	9.50 ​± ​2.12	1.787 (0.15)	8.00 ​± ​0.00	0.97 (0.407)	10.00 ​± ​2.83	1.324 (0.267)	45.50 ​± ​.71	0.674 (0.569)
	High school	74 (31.2)	9.36 ​± ​2.28		7.99 ​± ​2.17		6.69 ​± ​2.09		7.55 ​± ​1.99		6.92 ​± ​2.63		38.51 ​± ​8.01	
	College/university	137 (57.8)	8.80 ​± ​1.96		8.12 ​± ​1.99		7.18 ​± ​2.25		7.37 ​± ​1.74		6.84 ​± ​1.96		38.31 ​± ​7.55	
	Higher than graduate school	24 (10.1)	9.04 ​± ​2.31		8.08 ​± ​2.78		6.63 ​± ​2.72		6.83 ​± ​2.20		6.79 ​± ​2.43		37.38 ​± ​9.84	
Cancer history	Yes	11 (4.6)	9.27 ​± ​2.45	0.433 (0.666)	8.36 ​± ​2.11	0.453 (0.651)	7.45 ​± ​2.16	0.700 (0.484)	7.09 ​± ​1.14	−0.524 (0.601)	6.36 ​± ​2.38	−0.789 (0.431)	38.55 ​± ​7.97	0.089 (0.929)
	No	226 (95.4)	8.99 ​± ​2.09		8.07 ​± ​2.12		6.96 ​± ​2.27		7.39 ​± ​1.90		6.91 ​± ​2.24		38.33 ​± ​7.93	
Living with cancer survivors among family/acquaintances	Yes	116 (48.9)	8.79 ​± ​2.14	−1.516 (0.131)	7.71 ​± ​2.11	−2.688∗∗ (< 0.001)	6.61 ​± ​2.33	−2.528∗ (<0.05)	7.17 ​± ​1.95	−1.677 (0.095)	6.58 ​± ​2.44	−2.084∗ (<0.05)	36.86 ​± ​8.25	−2.852∗∗ (<0.01)
	No	121 (51.1)	9.21 ​± ​2.06		8.44 ​± ​2.07		7.35 ​± ​2.14		7.58 ​± ​1.78		7.18 ​± ​2.01		39.75 ​± ​7.34	

∗*P* < 0.05, ∗∗*P* < 0.01, ∗∗∗*P* < 0.001.

### 3.5 Attitudes regarding cancer survivors’ return to work based on general characteristics

In perceptions toward cancer survivors returning to work, men reported significantly more negative scores (*M* ​= ​29.33; *SD* ​= ​6.42) than women (*M* ​= ​26.26; SD ​= ​6.43) (*P* ​< ​0.001). Additionally, having a cancer survivor in the family or as an acquaintance significantly impacted participants’ perceptions of cancer survivors returning to work (*P* ​< ​0.001).

The statistical significance of the difference in acceptance of cancer survivors returning to work according to the characteristics of the participants was not verified (Table 6).

**Table 6:** Public attitudes toward cancer survivors regarding return to work according to general characteristics (*n* ​= ​237).

		*n* (%)	Perceptions (Mean ± SD)	*t/F* (*P*)	Acceptance (Mean ± SD)	*t/F* (*P*)
Gender	Male	120 (50.6)	29.33 ​± ​6.42	3.676∗∗∗ (<0.001)	17.10 ​± ​3.07	-0.862 (0.390)
	Female	117 (49.4)	26.26 ​± ​6.43		17.46 ​± ​3.23	
Age, years	20–29	58 (24.5)	26.6 ​± ​7.30	1.276 (0.283)	17.32 ​± ​3.43	2.404 (0.068)
	30–39	59 (24.9)	28.17 ​± ​7.03		16.49 ​± ​3.22	
	40–49	58 (24.5)	27.55 ​± ​6.23		17.25 ​± ​2.76	
	> 50	62 (26.2)	28.87 ​± ​5.69		18.01 ​± ​3.04	
Occupation	Professional/technical	58 (24.5)	27.64 ​± ​6.29	0.969 (0.408)	17.72 ​± ​2.96	1.428 (0.235)
	Office/administrative	63 (26.6)	27.43 ​± ​6.28		17.07 ​± ​3.06	
	Sales/Service worker	58 (24.5)	27.17 ​± ​7.55		17.65 ​± ​3.41	
	Production/Transportation/Labor	58 (24.5)	29.07 ​± ​6.18		16.68 ​± ​3.12	
Years of working	< 1	17 (7.2)	28.35 ​± ​5.80	0.943 (0.440)	18.05 ​± ​2.33	0.814 (0.518)
	1–3	48 (20.3)	26.23 ​± ​7.15		16.75 ​± ​4.12	
	3–5	21 (8.9)	28.43 ​± ​5.67		16.85 ​± ​2.43	
	5–10	59 (24.9)	28.51 ​± ​7.56		17.28 ​± ​3.02	
	> 10	92 (38.8)	27.97 ​± ​5.93		17.51 ​± ​2.92	
Shift pattern	Regular	163 (68.8)	27.79 ​± ​6.54	0.384 (0.765)	17.19 ​± ​3.03	0.457 (0.712)
	Contract	40 (16.9)	27.83 ​± ​7.03		17.22 ​± ​3.74	
	Daily/Part-time	16 (6.8)	29.25 ​± ​7.37		18.12 ​± ​2.75	
	Self-employment/employer	18 (7.6)	26.83 ​± ​5.58		17.50 ​± ​3.20	
Position	Staff	115 (48.5)	27.80 ​± ​7.08	0.668 (0.648)	17.15 ​± ​3.38	0.841 (0.522)
	Assistant manager	40 (16.9)	28.95 ​± ​6.60		17.87 ​± ​2.32	
	Manager	23 (9.7)	26.70 ​± ​6.00		17.13 ​± ​2.47	
	Deputy General manager/General manager	32 (13.5)	28.41 ​± ​5.92		16.65 ​± ​3.42	
	Director	9 (3.8)	25.78 ​± ​6.08		18.44 ​± ​3.74	
	Others (self-employment/employer)	18 (7.6)	26.83 ​± ​5.58		17.50 ​± ​3.20	
Staff numbers	1–49	116 (48.9)	27.40 ​± ​6.43	1.370 (.256)	17.22 ​± ​3.01	0.206 (0.814)
	50–299	65 (27.4)	27.48 ​± ​7.29		17.18 ​± ​3.63	
	300–4999	56 (23.6)	29.09 ​± ​6.01		17.51 ​± ​2.86	
Workplace	Indoor job	190 (80.2)	27.51 ​± ​6.60	−1.450 (0.148)	17.20 ​± ​3.06	−-0.811 (0.418)
	Outside job	47 (19.8)	29.06 ​± ​6.47		17.61 ​± ​3.49	
Monthly household income (USD)	< 1500	45 (19.0)	29.20 ​± ​6.85	2.192 (0.114)	17.35 ​± ​2.97	0.219 (0.804)
	1500–3000	142 (59.9)	27.11 ​± ​6.46		17.35 ​± ​3.23	
	> 3000	50 (21.1)	28.60 ​± ​6.57		17.02 ​± ​3.13	
Education	Less than middle school	2 (0.8)	29.00 ​± ​0.00	0.213 (0.887)	20.50 ​± ​3.53	1.850 (0.139)
	High school	74 (31.2)	28.16 ​± ​6.68		17.55 ​± ​3.21	
	College/university	137 (57.8)	27.76 ​± ​6.36		16.95 ​± ​3.07	
	Higher than graduate school	24 (10.1)	27.00 ​± ​8.00		18.04 ​± ​3.20	
Cancer history	Yes	11 (4.6)	27.18 ​± ​4.83	−-0.327 (0.744)	15.90 ​± ​3.56	−1.483 (0.139)
	No	226 (95.4)	27.85 ​± ​6.67		17.34 ​± ​3.12	
Living with cancer survivors among family/acquaintances	Yes	116 (48.9)	26.22 ​± ​6.73	−3.745∗∗∗ (<0.001)	17.32 ​± ​3.37	0.214 (0.831)
	No	121 (51.1)	29.35 ​± ​6.10		17.23 ​± ​2.94	

∗*P* < 0.05, ∗∗*P* < 0.01, ∗∗∗*P* < 0.001.

## 4 Discussion

This study is the first to investigate the combination of attitudes of workers toward cancer and patients with cancer and cancer survivors' return to work. In particular, the results were significant because the sample was recruited through stratified recruitment; therefore, the age and occupational groups were evenly distributed. To facilitate cancer survivors' successful adaptation to the community, the public must understand cancer survivors and actively accept their unique situation. A nonpositive public attitude could lead to the exclusion of certain groups from social participation and lead to negative decision-making and alienation from society.^7^ According to some relevant data from 2013, 46.8% of cancer survivors in Korea changed their employment status (eg, taking a leave of absence or facing job loss after cancer diagnosis), and 84.1% of them lost their jobs.^23^ Furthermore, in Korea, the job turnover rate attributable to cancer is about 50%, and the probability that cancer survivors will be reemployed after retirement is half of that of the general public.^20^ Influencing these results may be a more important factor in shaping society's perceptions regarding patients with cancer rather than individual work competency.^11^^,^^24^ Therefore, on the basis of the current study results, we intend to discuss Korean workers' perceptions regarding cancer survivors' return to work.

Compared to the results of Cho et al, ^10^ we find that the Korean general public's perceptions regarding cancer and patients with cancer is changing positively. The most positive sub-domain in this study was discrimination, indicating that the degree of discrimination against patients with cancer was low. Therefore, it seems that patients with cancer are not too reluctant to disclose that they have been diagnosed with cancer. The most negative perception was tied to cancer recovery. These results showed that despite improvements in cancer treatments and prognosis due to advances in medical technology and the fact that the 5-year relative survival rate for cancer has exceeded 70% and is rising every year,^25^ positive perceptions regarding cancer treatment are not very high. These results are similar to those of a study by Cho et al, ^10^ which used the same tool to assess the attitudes of the general public, where 58% answered negatively. This shows that the general public still has a negative perception of the possibility of cancer treatment.

These results must be considered in the same context as the results of previous studies^24^^,^^26^^,^^27^—that is, we must acknowledge that cancer survivors experience social prejudice when returning to society. However, answers to the item “It is impossible to treat cancer regardless of highly developed medical science” differ among nursing students^28^ and medical personnel.^29^ The workers who participated in this study expressed more negative perceptions than nursing students and medical personnel. This difference may be attributable to the difference in information acquisition between medical personnel, who can access accurate medical information more easily, and the general public; there is also some difference between medical personnel, who encounter actual patients in clinical practice, and students. The differences between the public and medical professionals show the necessity of finding a more active and popular way to easily disseminate accurate information about cancer and cancer treatment to the general public.

Compared to the results of Shim et al, ^21^ this study shows that perceptions and acceptance of cancer survivors' return to work appear to be improving. Furthermore, these findings may indicate an increasingly positive change in our society regarding cancer survivors’ return to work. Nevertheless, symptoms such as pain, memory loss, depression, and low energy are considered negative factors for reinstatement. This negative perception is complemented by the perception that cancer survivors have low self-confidence and less are less dedicated to their work, which may lead to the perception that it is desirable to replace them with healthy employees. Although many workers do not hold negative perceptions regarding cancer survivors, they seem to show a difference in their perceptions related to work (eg, work efficiency). A similar trend was observed with regard to overall positive perceptions in “acceptance of cancer survivors returning to work.” This overall positive result may be interpreted as concealing the perception that cancer survivors cannot demonstrate the necessary labor productivity required of a worker and that they cannot achieve better results without the aid of healthy workers.

Such a presumption is supported by the fact that the number of cancer survivors returning to work is considerably lower in Korea than in foreign countries. In Korea, 47%–53% of cancer survivors change jobs because of their cancer diagnosis. Furthermore, the likelihood of cancer survivors returning to work is 47% lower than that of the general population.^20^ Only 30.5% of cancer survivors in Korea return to work^20^; this figure is lower than that in foreign countries, where 60–92% of cancer survivors return to work.^16^

With regard to perceptions of cancer, cancer survivors, and perceptions of cancer survivors' reinstatement, men showed a more negative attitude than women. The results seem to indicate that men perceived cancer survivors to be socially weaker (compared to women's corresponding perceptions) and felt that they could not make a significant contribution to society. This supports the results of previous studies that men tend to be more stigmatizing toward—and biased against—the socially disadvantaged (eg, the elderly and the physically disabled) compared to women.^30^

Furthermore, among the subfactors of perceptions regarding cancer and cancer survivors, there was a difference in perceptions based on occupation in determining the economic feasibility of cancer survivors as well as perceptions regarding their reinstatement. It was observed that the perceptions of manual laborers were the most negative.^31^ A previous study indicated that cancer survivors in manual labor occupations may find it difficult to keep a job because they experience after-effects and general physical weakness during their cancer diagnosis and treatment.^32^ Physically demanding work seemed to negatively impact cancer survivors' ability to return.^31^ These previous findings support the results of this study. This may explain why people in the professional subset displayed greater negative perceptions toward cancer survivors' return to work than people in other occupations. According to a report on the return to work status of patients with cancer in Korea, manual laborers who had cancer were more likely to lose their jobs compared to white-collar workers^20^; as soon as they received their cancer diagnosis, they either received a recommendation to resign or were physically removed from the workplace. One such worker described quitting the job because of the heavy physical burden.^32^ Furthermore, the finding that there are some differences in perceptions between genders seems to be attributable to the cultural characteristics of Korean society, where men are expected to be highly responsible for their economic activities.

Finally, study participants with cancer survivors as family members and acquaintances showed more positive perceptions than participants without such connections. This finding is similar to that of previous research regarding interpersonal contact with disabled or elderly people; it is known to have a positive effect on perceptions of the socially disadvantaged.^30^^,^^33^^,^^34^ In short, the current results can be considered in the same context as previous results,^35^ which have indicated that a prior knowledge and experience of others is conducive to building an understanding of differences in others instead of encouraging discrimination. The current study results could indicate that Korean workers' perceptions regarding cancer survivors and their return to work are gradually improving. Nevertheless, accurate information and support are necessary for cultivating a more positive perception toward cancer survivors’ return to work; furthermore, it should be noted that more policies and education are necessary to achieve this purpose.

### 4.1 Limitations and suggestions

Our study had several limitations. Our sampled subjects did not show a realistic and truly representative distribution of Korean workers; as this study is not a large-scale epidemiologic study, it is difficult for the number of subjects to represent the current status of all workers in Korea. Therefore, it is difficult to generalize the study's findings as the overall perception of all Korean workers. Furthermore, participants may have reported more positive attitudes toward cancer and cancer survivors and stronger openness toward disclosing cancer diagnosis because of the need for social desirability; this, in turn, may have resulted in an underestimation of any actual negative attitudes toward cancer. In addition, the study results may be culture-specific to Korea, and the findings may, therefore, not be generalizable to other countries. Despite these limitations, this study provides significant findings that offer insight into attitudes toward patients with cancer and survivors' return to work in Korea.

Future research should examine the various factors associated with cancer stigma, which may pose barriers to return to work of patients with cancer. Most importantly, negative perceptions regarding cancer survivors’ return to work can negatively affect the national economy and individual quality of life; thus, there is a strong need for a more systematic approach to cancer advocacy.

## 5 Conclusions

This study is the first study to investigate and recruit workers evenly by age and occupational group through a stratified expression. The results of this study and significant variables, such as gender and life experience with patients with cancer, can be used as basic data for policy-making and development of social awareness improvement programs necessary for cancer survivors' return to daily life and work.

## CRediT author statement

**Si Eun Lee**: Conceptualization, Data collection, Formal analysis, Investigation, Writing – Original Draft; **Eun Young Park**: Conceptualization, Formal analysis, Investigation, Writing – Original Draft. All authors had full access to all the data in the study, and the corresponding author had final responsibility for the decision to submit for publication. The corresponding author attests that all listed authors meet authorship criteria and that no others meeting the criteria have been omitted.

## Declaration of competing interest

The authors declare no conflict of interest.

## Funding

This work was financially supported by the Korean Oncology Nursing Society.

## Ethics statement

The study was approved by the Institutional Review Board of the Gachon University (IRB No. 1044396-202201-HR-017). All participants provided written informed consent.

## Data availability statement

The data that support the findings of this study are available upon request from the corresponding author, Park. The data are not publicly available due to restrictions, for example, their containing information that could compromise the privacy of research participants.
